# Application value of three-dimensional arterial spin labeling perfusion imaging in investigating cerebral blood flow dynamics in normal full-term neonates

**DOI:** 10.1186/s12887-019-1876-x

**Published:** 2019-12-13

**Authors:** Jia-Ning Wang, Jia Li, Huai-Jun Liu, Xiao-Ping Yin, Huan Zhou, Ya-Ting Zheng, Na An, Si Liang, Zuo-Jun Geng

**Affiliations:** 1 Department of Radiology, The Second Hospital of Hebei Medical University, 215 Heping West Road, Shijiazhuang, 050005 China; 2 Department of Otolaryngology Head and Neck Surgery, Baoding Second Hospital, Baoding, 071000 China; 3 Department of Radiology, Affiliated Hospital of Hebei University, Baoding, 071000 China; 4 Department of Pediatric, Affiliated Hospital of Hebei University, Baoding, 071000 China; 5 Department of Anesthesiology, Affiliated Hospital of Hebei University, Baoding, 071000 China

**Keywords:** Neonate, Arterial spin labeling, Perfusion imaging

## Abstract

**Background:**

This study aims to investigate the application value of three-dimensional arterial spin labeling (3DASL) in investigating cerebral blood flow dynamics in full-term neonates.

**Methods:**

A total of 60 full-term neonates without known intracranial pathology were recruited for 3DASL examination. These neonates were divided into three groups: 1–3 day group, 4–7 day group, and 8–15 day group. On the cerebral blood flow (CBF) images, regions of interest (ROI) were selected from the frontal white matter, parietal white matter, basal ganglia, corona radiata, thalamus and brainstem, and the CBF values of each ROI were recorded. The CBF values of ROIs at bilaterally symmetric locations, the values of each ROI between males and females, and the values of each ROI among these three different age groups were compared.

**Results:**

The difference in CBF values of the frontal white matter, parietal white matter, basal ganglia, corona radiata and thalamus at the bilateral symmetric positions were not statistically significant. There was no statistical difference in the CBF values of each brain region between the male and female groups. The CBF values at the basal ganglia region, corona radiata and parietal white matter were higher in the 8–15 day group, when compared to the 1–3 day and 4–7 day groups (*P* < 0.05). The CBF value at the basal ganglia region was higher in the 4–7 day group, when compared to the 1–3 day group (*P* < 0.05). The CBF value at the frontal white matter was lower in the 4–7 day group, when compared to the 1–3 day and 8–15 day group (*P* < 0.05). The CBF value at the brainstem was higher in the 4–7 day group, when compared to the 1–3 day and 8–15 day groups (*P* < 0.05).

**Conclusion:**

The 3DASL can quantitatively measure CBF, and be used to evaluate cerebral hemodynamics in neonates. The basal ganglia region and corona radiata CBF increases with the increase in neonatal diurnal age.

## Background

Arterial spin labeling (ASL) uses magnetically labeled arterial blood as an endogenous contrast agent, and uses water molecules as a free diffused internal tracer. It first induces reverse blood spin labels on the imaging plane. When blood flows to the imaging slices and exchange with tissues, the signals are reduced and images are produced and collected [1–3]. According to different labeling methods, ASL can be classified into continuous arterial spin labeling (CASL) and pulsed arterial spin labeling (PASL) [4].

Three-dimensional arterial spin labeling (3DASL) imaging is a functional magnetic resonance imaging (MRI) that reflects cerebral blood perfusion, the continuous labeling of arterial blood flow in the brain, and three-dimensional (3D) rapid imaging of the whole brain and measurement of cerebral blood flow (CBF) changes after the labeling of blood flow in brain tissues [5]. This technique has the following advantages: non-invasiveness, no need to inject the contrast agent, high safety, and its application to the neonatal brain perfusion imaging method [6]. At present, 3DASL has been widely used in central nervous system diseases, but studies on the value of 3DASL technology have mostly focused on adults, and few studies have been conducted on neonates [7, 8]. Before the 3DASL sequence was used to analyze diseases, it was crucial to understand the hemodynamic performance of this sequence in normal human brains.

At present, reports on cerebral hemodynamics in full-term neonates are rare. The aim of the present study was to investigate the characteristics of cerebral perfusion in full-term neonates, and the impacts of different age groups on cerebral perfusion in full-term neonates.

## Methods

### Study design and participants

From December 2016 to March 2018, the data of 60 full-term neonates without known intracranial pathology (34 males and 26 females, chronological age of 2–15 days), who received head MRI scans and 3DASL examinations due to neonatal jaundice, poor feeding and other diseases, did not receive cerebral perfusion imaging, and needed to stay in the hospital for observation, were collected. Newborns with abnormal brain development or intracranial hemorrhage due to birth injury were excluded. “Full term” means a gestational age ≥ 37 weeks. Inclusion criteria: (1) Patients who were full-term neonates; (2) newborns who finished the skull MRI scan; (3) patients who had no abnormalities revealed by the skull MRI scan. These full-term neonates were divided into three groups according to the MRI scan: chronological age: 1–3 day group, 19 neonates (11 neonates in the male group and eight neonates in the female group); 4–7 day group, 21 neonates (12 neonates in the male group and nine neonates in the female group); 8–15 day group, 20 neonates (11 neonates in the male group and nine neonates in the female group). The present study was approved by the hospital ethics committee, and all neonatal family members provided a signed informed consent.

### Inspection methods

All data were collected using a GE Discovery 750B 3.0 T magnetic resonance scanner, and the acquisition coil was a 16-channel coil. The scanning sequences included T1WI, T2WI, DWI, T2-flair and 3DASL imaging. The 3DASL sequence parameters: repetition time (TR) = 4376 ms, echo time (TE) =11 ms, flip angle = 111°, field of view (FOV) = 16 × 16 cm, thickness = 4.0 mm, and excitation = three times. T1WI parameters: TR = 540 ms, TE = 10 ms, FOV = 16 × 16 cm, and slice thickness = 4.5 mm. T2WI parameters: TR = 3880 ms, TE = 85 ms, FOV = 16 × 16 cm, and slice thickness = 4.5 mm. T2-flair parameters: TR = 9000 ms, TE = 90 ms, FOV = 16 × 16 cm, and slice thickness = 4.5 mm. The 3D-ASL was a 3D-PCASL, which was realized through the 3D-FSE sequence, and this has a single delay time. For pulsed laser deposition (PLD), 1025 ms was chosen for the following reasons: first, newborn babies have rapid blood flow; second, the marked position is relatively short from the collection position (small head, short relative distance); third, it has a short PLD time and good signal strength, because newborn babies have thin blood vessels.

The pediatrician gave an intramuscular injection of phenobarbital at a dose of 5 mg/kg in the ward at 15–20 min before the scan. After the patient fell asleep, the patient was carried to the MRI room for examination. The patient was placed in the supine position, and the head was placed safely in the head coil for fixation. A pediatric nurse was present during the examination.

### Data processing

The CBF plots were obtained using a GE AW 4.6 workstation and the FunctooI software. CBF = (Mean arterial pressure [MAP]-Intracranial pressure [ICP]) /Cerebrovascular resistance (CVR). The CBF values in the area of interest were measured. The regions of interest (ROI) of all patients included the bilateral basal ganglia, thalamus, corona radiata, frontal and parietal white matter and brain stem. ROI was approximately 50 square millimeters. In order to ensure the consistency and comparability of the ROI, the mirror copy ROI method was adopted for measuring each brain area. Each brain area was measured for three times and averaged. Attempts were made to ensure that all newborns had the same ROI and the same shape. The level of ROI was determined according to the anatomical mark, and the consistency of the level was ensured.

### Statistical analysis

**Fig. 1:**
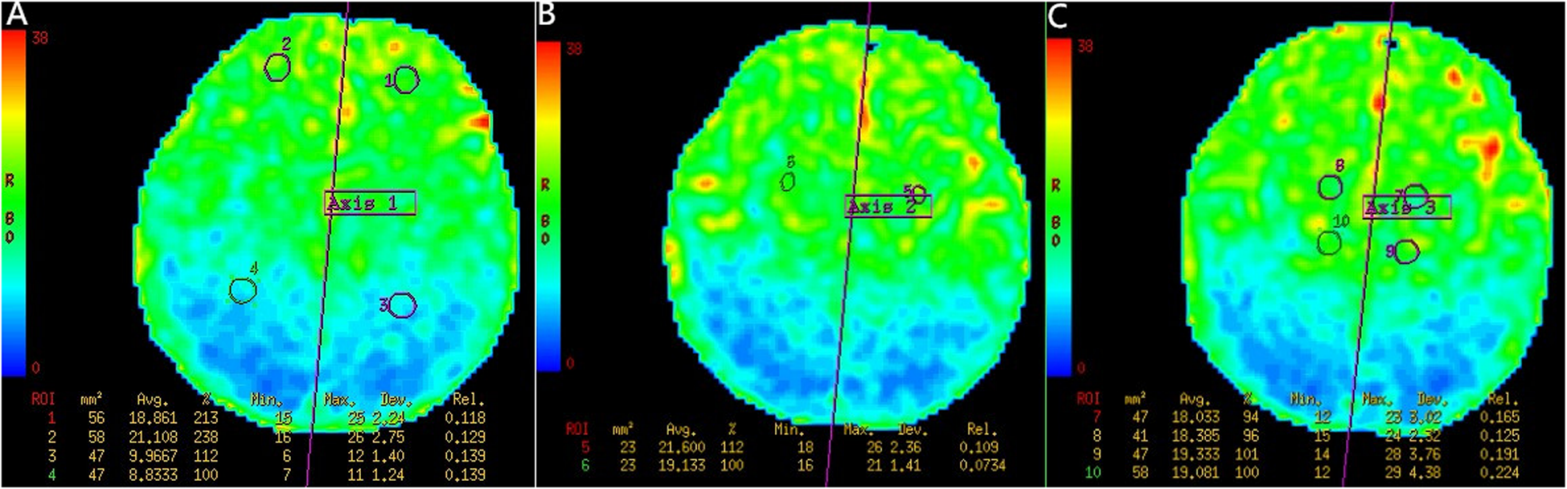
A female, 6 days old, and full-term. **a**-**c** present the CBF diagrams that show the cerebral hemisphere cerebral blood flow perfusion bilateral symmetry: ROIs 1 and 2, for the frontal white matter; ROIs 3 and 4, for the parietal white matter; ROIs 5 and 6, for the corona radiata; ROIs 7 and 8, for the basal ganglia; ROIs 9 and 10, for the thalamus. The purple line represents the central axis of the symmetry. The color bars on the left in red, yellow, green and blue indicate the perfusion from high to low

**Fig. 2:**
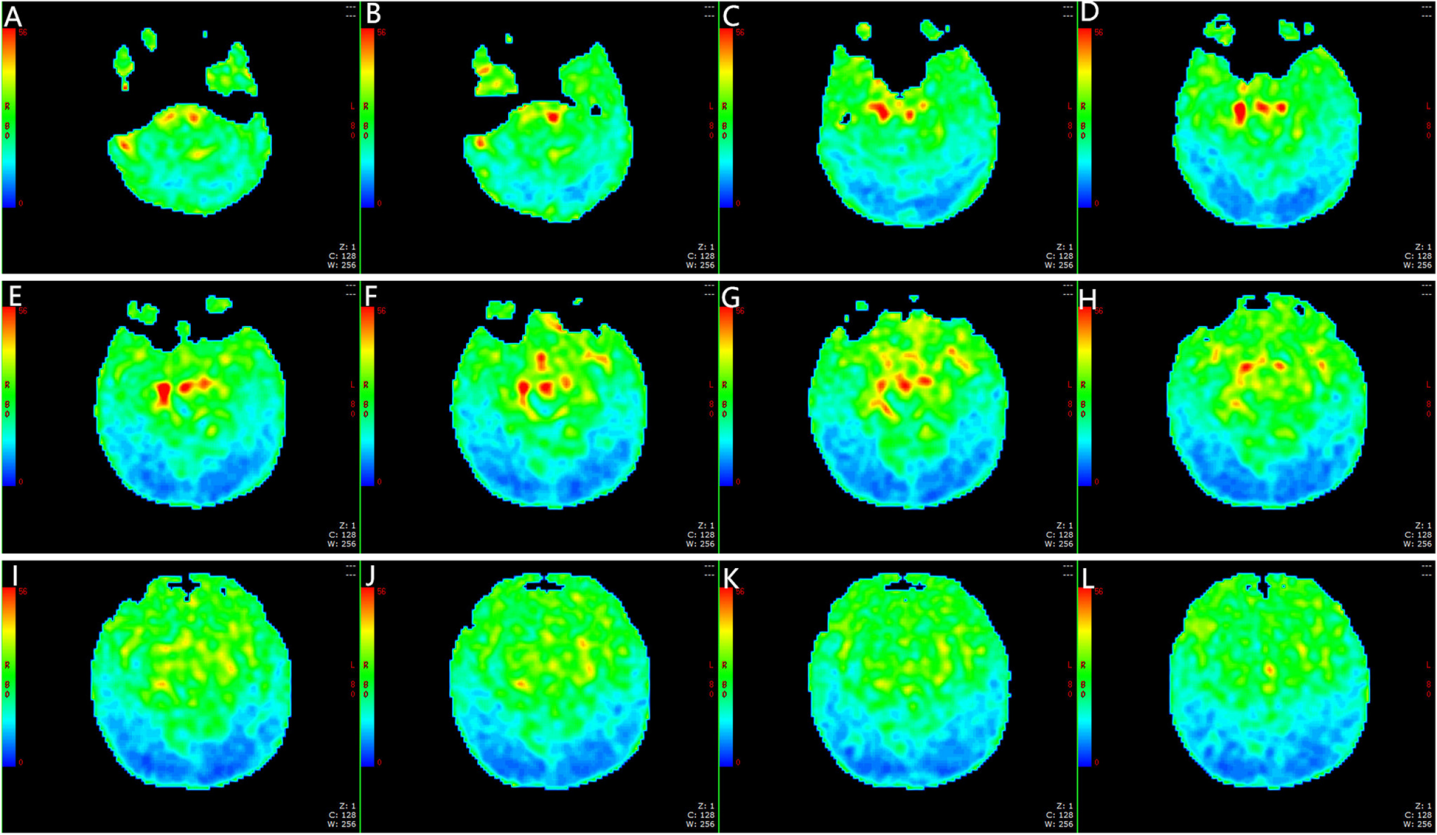
A male, 2 days old, and full-term. **a**-**l** present the CBF charts. No obvious abnormal perfusion could be observed in the CBF diagram

SPSS statistics 17.0 was used for the data analysis, and the data were expressed as mean ± standard deviation. For multiple comparisons, each value was compared using one-way analysis of variance (ANOVA) following the Dunnett’s test, when each datum was normally distributed, while non-normally distributed continuous data were compared using non-parametric tests. Counting data were analyzed using Chi-square test. The data that needed to be compared were tested for normal distribution and homogeneity of variance. The difference in CBF values in the symmetrical areas of the bilateral brain regions was determined by paired *t*-test. The CBF values for males and females were compared by independent sample *t*-test. ANOVA was used to determine the differences in CBF value among age groups, and *P* < 0.05 was considered statistically significant (Figs. 1 and 2).

## Results

The non-enhanced MRI scan and 3DASL of 60 full-term neonates without known intracranial pathology revealed no clear abnormalities. The 3DASL diagram could clearly show the different blood flow signals in each brain region, which were bilaterally symmetric, and the visual cortex blood flow signals were higher than those in the white matter. The CBF values of the basal ganglia, brainstem, thalamus and corona radiata were higher than those of the white matter in the frontal lobe and parietal lobe. However, there was no statistically significant difference in CBF values in the same area between both sides (*P* > 0.05, Table 1).

**Table 1:** comparison of CBF values of ROI in each symmetric brain region (mean ± standard deviation) (ml/100 g/min)

	*basal ganglia*	*thalamus*	*corona radiata*	*frontal white matter*	*parietal white matter*
*Left*	*31.59 ± 6.53*	*28.80 ± 5.61*	*24.76 ± 4.58*	*22.47 ± 3.64*	*11.23 ± 2.31*
*Right*	*29.87 ± 7.00*	*28.34 ± 6.43*	*23.34 ± 4.98*	*22.78 ± 3.36*	*11.26 ± 2.12*
*t*	*1.38*	*0.42*	*1.63*	*0.48*	*0.08*
*P*	*0.17*	*0.67*	*0.11*	*0.63*	*0.94*

The CBF values of ROIs in various brain regions between male and female genders were compared. The CBF values of the basal ganglia region, corona radiata, hypothalamus and brainstem were slightly higher in females than in males, while the CBF values of the frontal and parietal white matter were slightly higher in males than in females. However, the differences in CBF values in ROIs in the bilateral basal ganglia, thalamus, corona radiata, frontal lobe and parietal lobe white matter, and brain stem between males and females were not statistically significant (*P* > 0.05, Table 2).

**Table 2:** comparison of CBF values in neonates of different genders (mean ± standard deviation) (ml/100 g/min)

	*basal ganglia*	*thalamus*	*corona radiata*	*frontal white matter*	*parietal white matter*	brain stem
male(*N* = 34)	*29.95 ± 6.52*	*27.36 ± 5.63*	*23.59 ± 4.80*	*22.92 ± 3.71*	*11.36 ± 2.08*	*33.64 ± 7.06*
female(*N* = 26)	*31.33 ± 5.84*	*29.64 ± 4.98*	*24.70 ± 2.96*	*22.37 ± 2.87*	*10.94 ± 1.29*	*34.93 ± 4.35*
*t*	*0.85*	*1.63*	*1.04*	0.63	0.91	0.82
*P*	*0.40*	*0.19*	*0.30*	0.53	0.37	0.41

Note: The CBF values of basal ganglia, thalamus, radial crown, frontal white matter and parietal white matter were the bilateral average values

In comparing the CBF values in various regions among the three different age groups, the basal ganglia region, corona radiata, and parietal white matter CBF values were higher in the 8–15 day group, when compared to the 1–3 day and 4–7 day groups (*P* < 0.05). The basal ganglia region CBF values were higher in the 4–7 day group, when compared to the 1–3 day group (*P* < 0.05). The frontal white matter CBF values were lower in the 4–7 day group, when compared to the 1–3 day and 8–15 day groups (*P* < 0.05). The brainstem CBF values were higher in the 4–7 day group, when compared to the 1–3 day and 8–15 day groups (*P* < 0.05). However, there was no statistically significant difference in thalamic CBF among the three groups (Table 3).

**Table 3:** comparison of CBF values in each brain region of the three groups at different ages (mean ± standard deviation) (ml/100 g/min)

	*basal ganglia*	*thalamus*	*corona radiata*	*frontal white matter*	*parietal white matter*	brain stem
1-3d(*N* = 19)	*25.93 ± 3.39*	*27.15 ± 5.68*	*22.58 ± 3.54*	*23.154 ± 3.75*	*10.767 ± 1.43*	*31.54 ± 4.06*
4-7d(*N* = 21)	30.26 ± 5.96*	*28.57 ± 6.08*	*22.67 ± 3.85*	21.03 ± 2.18*	*10.66 ± 1.37*	37.22 ± 6.49*
8-15d(*N* = 20)	35.24 ± 5.24*^#^	*29.24 ± 4.45*	26.97 ± 3.41*^#^	*23.96 ± 3.40*^*#*^	12.12 ± 2.11*^#^	*33.54 ± 5.93*^*#*^
*F*	*16.78*	*0.74*	*9.66*	*4.71*	*4.79*	*5.28*
*P*	*< 0.01*	*0.48*	*< 0.01*	*0.01*	*0.01*	*0.01*

Note: The CBF values of basal ganglia, thalamus, radial crown, frontal white matter and parietal white matter were the bilateral average values. * means that compared with the 1–3 day group, *P* < 0.05; # means that compared with compared with the 4–7 day group, *P* < 0.05

## Discussion

In the present study, the 3DASL sequence used was for whole-brain volumetric perfusion imaging, which can effectively overcome the magnetic sensitivity artifacts and motion artifacts brought by the echo-planar imaging (EPI), in order to obtain high-resolution images. ASL does not need an injection of a contrast agent, and can be repeatedly performed in the short term [9]. Recently, 3DASL has been widely used in diagnosing central nervous system diseases among adults. In particular, it can be widely used in diagnosing diseases of the central nervous system in newborns, such as neonatal hypoxic-ischemic encephalopathy (HIE) [10], premature white matter injury, hypoglycemic encephalopathy, etc. The eventual potential value of these sequences is for HIE, white matter injury, etc. Therefore, full-term neonates were included.

In the present study, the CBF values were evaluated by bilateral comparison. The ROIs were selected in the right basal ganglia region, thalamus, corona radiata, white matter in frontal lobe and white matter in parietal lobe, while the left ROIs were automatically selected through the symmetry axis. The results revealed that there was no statistically significant difference in CBF values among the bilateral symmetrical brain regions, which is consistent with previous studies on adults [11]. The CBF values measured by ASL greatly varies among individuals [12, 13]. Hence, the left-right comparison is a practical method for determining disease status.

In the present study, the CBF values of the basal ganglia, corona radiata, thalamus and brainstem were higher, when compared to those of the white matter in the frontal lobe and parietal lobe. However, the increase in CBF values had no statistically significant difference. Domestic and foreign literature reports on cerebral blood perfusion in adult human brains have revealed that female cerebral blood perfusion is higher, when compared to that in males [14–17]. The CBF values of the basal ganglia area, brainstem, thalamus and corona radiata were slightly higher in the female group, when compared to the male group, and the CBF values of the white matter in the frontal lobe and parietal lobe were slightly higher in the male group, when compared to the female group, but the differences were not statistically significant. This is inconsistent with previous reports, but is consistent with some reports on adults in China. The first reason for this result may be that the CBF value of female neonates is indeed higher than that of male neonates. However, the sample size in the present study was small, and there was no significant statistical difference. The second possibility is that the difference in CBF value between male and female neonates is indeed not as significant as that between male and female adults, and the differences in estrogen level [18], hematocrit [19] and blood viscosity [20] between male and female neonates are not as significant as that among adults.

In the present study, 60 neonates were divided into three groups. The results revealed that the CBF values of the basal ganglia area, corona radiata and parietal lobe white matter were higher in the 8–15 day group, when compared to those in the 1–3 day and 4–7 day groups (all, *P* < 0.05), and the CBF values of the basal ganglia area were higher in the 4–7 day group, when compared to the 1–3 day group (all, *P* < 0.05). These supports that the CBF values of these brain areas exhibit an upward trend with the increase in age. A recent gadolinium bolus-tracking study in young children revealed that perfusion increased with age, supporting the link between perfusion and synaptic density, which also increases during childhood [21]. Subcortical white matter and watershed are more sensitive to chronic hypoxic ischemia [22, 23]. The decrease in CBF in adults with age is caused by brain atrophy [24, 25], while the brain tissue of newborns presents a process of rapid growth. With the increase in age, the metabolism and oxygen consumption in the brain increases, which can increase CBF. Hence, in the clinical application of neonatal brain ASL, the influence of age on CBF values should be considered.

Limitations of the present study: (1) The sample size was small, the range of daily age covered by the sample was narrow, and merely newborns of 1–15 days old were collected. In the future, the sample size and range of daily age would be expanded for research. (2) The choice of ROI was subject to the subjective influence of the operators. The blood vessels and cerebral sulci of newborns could not be completely avoided when selecting the ROI. The method of taking the average value of three times of the measurement was adopted to ensure the reliability of the data, as far as possible. (3) The influence of CBF maps on the anatomical reference should be further researched. (4) A comparison of whole brain CBF values between different groups was lacking, and should be further evaluated. (5) The PLD used in the present study appeared to be shorter than the ASL white paper. Blood in the vessels could show better signals, but the measured signals were not perfused, which should be further researched. (6) The pixel of the ASL was usually larger than 3 × 3 mm^2^. The manually selected ROI (50 mm^2^) only has 4–5 pixels, which is an unstable measurement, and should be further researched.

## Conclusion

In summary, ASL has the characteristics of noninvasiveness and simplicity, and has unique advantages in the clinical application of neonates. In clinical application, gender has no significant effect on the CBF value. Hence, the effect of age on the CBF value should be considered, and a bilateral comparison method should be adopted.

## Data Availability

All data generated or analysed during this study are included in this published article.

## Abbreviations

3D: Three-dimensional
3DASL: Three-dimensional arterial spin labeling
ASL: Arterial spin labeling
CASL: Continuous arterial spin labeling
CBF: Cerebral blood flow
EPI: Echo-planar imaging
MRI: Magnetic resonance imaging
PASL: Pulsed arterial spin labeling
ROI: Regions of interest
